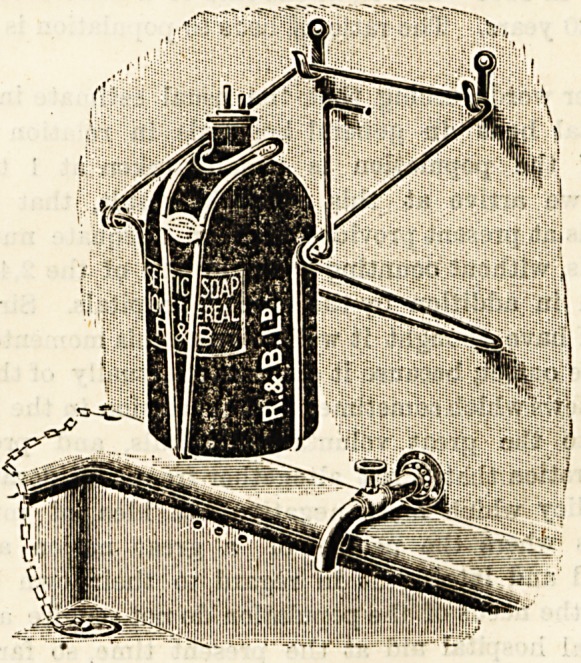# New Appliances and Things Medical

**Published:** 1903-03-07

**Authors:** 


### NEW APPLIANCES AND THINGS MEDICAL.

### SOAP BOTTLE CRADLE.

(Reynolds and Branson, Ltd., 13 Briggate, Leeds.)

Our illustration explains the mechanism of a very ingenious device for supplying surgeons and others with a reliable antiseptic soap. The swing cradle, which may be at pleasure hung on the wall, or stood on a ledge over the washing basin, contains a bottle for the reception of a liquid antiseptic soap. The bottle is tilted and the soap issues in a small steady stream when the finger is pressed on a small lever. In this way the bottle is never contaminated by contact with soiled fingers, and there is no waste of soap. We regard this soap cradle as a very useful adjunct to the up-to-date operating theatre.

## Figures and Tables

**Figure f1:**
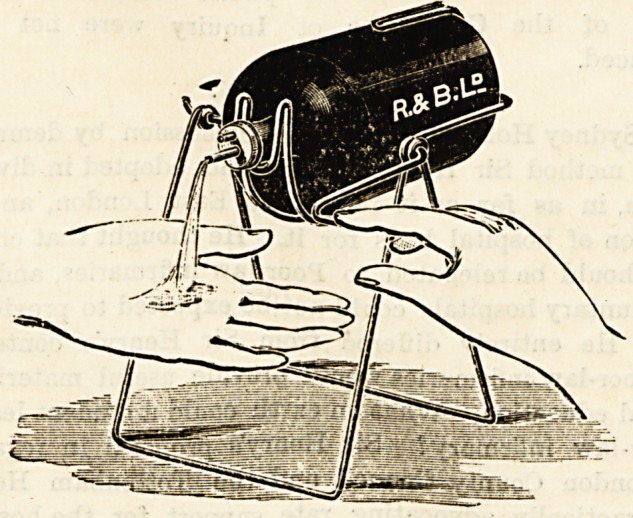


**Figure f2:**